# Curative resection of borderline resectable perihilar cholangiocarcinoma following neoadjuvant gemcitabine and cisplatin plus durvalumab immunochemotherapy: a case report

**DOI:** 10.1093/jscr/rjag255

**Published:** 2026-04-15

**Authors:** Shogo Tanaka, Hiroshi Tsukuda, Takuma Okada, Akihiro Tanaka, Ryugo Sawada, Kiyotaka Yukimoto, Yutaka Tamamori, Yuichi Fumimoto, Shinsuke Hiramatsu, Yoshio Ohta

**Affiliations:** Department of Hepato-Biliary-Pancreatic Surgery, Izumi City General Hospital, 4-5-1 Wakecho, Izumi 594-0073, Japan; Department of Oncology, Izumi City General Hospital, 4-5-1 Wakecho, Izumi 594-0073, Japan; Department of Hepato-Biliary-Pancreatic Surgery, Izumi City General Hospital, 4-5-1 Wakecho, Izumi 594-0073, Japan; Department of Oncology, Izumi City General Hospital, 4-5-1 Wakecho, Izumi 594-0073, Japan; Department of Gastroenterological Surgery, Izumi City General Hospital, 4-5-1 Wakecho, Izumi 594-0073, Japan; Department of Gastroenterological Surgery, Izumi City General Hospital, 4-5-1 Wakecho, Izumi 594-0073, Japan; Department of Gastroenterological Surgery, Izumi City General Hospital, 4-5-1 Wakecho, Izumi 594-0073, Japan; Department of Gastroenterological Surgery, Izumi City General Hospital, 4-5-1 Wakecho, Izumi 594-0073, Japan; Department of Gastroenterology, Izumi City General Hospital, 4-5-1 Wakecho, Izumi 594-0073, Japan; Department of Diagnostic Pathology, Izumi City General Hospital, 4-5-1 Wakecho, Izumi 594-0073, Japan

**Keywords:** biliary tract cancers, borderline resectable, complete response, immunochemotherapy

## Abstract

Postoperative survival benefits of neoadjuvant gemcitabine and cisplatin plus durvalumab (GCD) immunochemotherapy for biliary tract cancers, which meet the criteria for borderline resectability, remain limited. A 75-year-old male presented to our hospital with epigastric pain. Diagnostic imaging demonstrated perihilar cholangiocarcinoma invading the right hepatic artery with lymph node metastasis. The patient was diagnosed with borderline resectable perihilar cholangiocarcinoma (cT3N1M0 Stage IIIC) and treated with seven cycles of GCD immunochemotherapy. Dynamic computed tomography post-immunochemotherapy revealed that the tumor decreased in size, and arterial invasion and swollen lymph nodes had disappeared. At 6 months following immunochemotherapy initiation, the patient underwent left hepatectomy, lymph node dissection, and biliary reconstruction. Histopathology showed inflammatory cell infiltration in the bile duct mucosa without cancer cells. No lymph node metastases were detected. At 2 years postoperatively, the patient remains stable without recurrence.

## Introduction

In 2022, a Phase 3 study (TOPAZ-1 trial) revealed that gemcitabine and cisplatin plus durvalumab (GCD) immunochemotherapy resulted in longer overall survival (OS) and progression-free survival (PFS) with higher objective response rates (ORRs) than conventional GC chemotherapy for advanced or unresectable biliart tract cancers (BTCs) [1], rendering it the first-line treatment to date. Moreover, evidence on the effectiveness of neoadjuvant GCD immunochemotherapy for borderline resectable BTCs (technically resectable but with significant vascular involvement or high biological risk factors, including high carbohydrate antigen 19–9 [CA19–9] levels [>500 U/ml], and lymph node involvement) is lacking [2].

We herein report a case in which the patient underwent resection and achieved complete remission (CR) following GCD immunochemotherapy for borderline resectable perihilar cholangiocarcinoma.

## Case report

**Figure 1:**
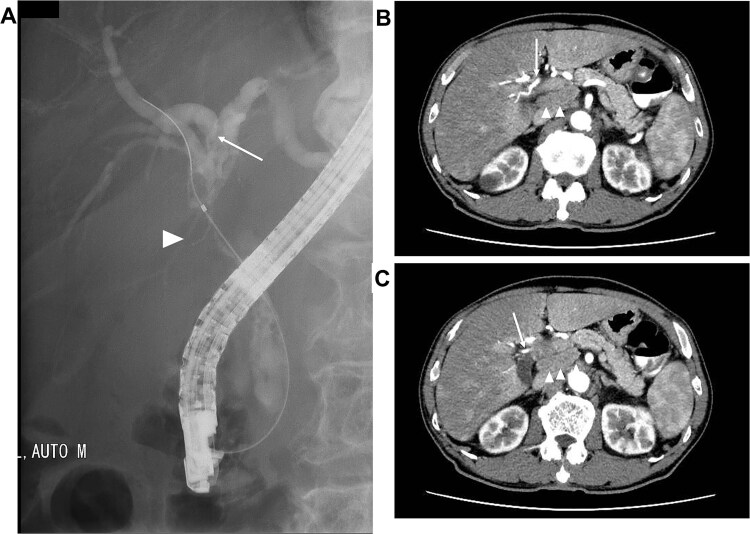
Images before neoadjuvant GCD immunochemotherapy (A) ERC revealing biliary stricture extending from the CHD (arrow head) to the hepatic hilum. Right posterior branch (arrow) joins the left hepatic duct. (B and C) dynamic CT demonstrating the tumor invades the RHA (arrow), and the lymph node surrounding the common hepatic artery and retropancreatic area is swollen (arrow head).

A 75-year-old male presented to our hospital owing to recurrent epigastralgia. Plain computed tomography (CT) revealed slight intrahepatic bile duct dilation and common hepatic duct (CHD) stenosis. Laboratory test results demonstrated elevated serum concentrations of alanine aminotransferase (49 U/ml), γ-glutamyl transpeptidase (287 U/ml), and CA19–9 (52.0 U/ml). Endoscopic retrograde cholangiography (ERC) revealed CHD stenosis and irregular wall at the confluence of the right anterior and posterior bile duct to the left hepatic duct (Fig. 1A). Biopsy of the stenosed CHD revealed adenocarcinoma. Dynamic CT revealed a 2-cm-diameter mass consistent with CHD stenosis, suggesting right hepatic artery (RHA) invasion (Fig. 1B). Furthermore, a swollen lymph node around the common hepatic artery and the retropancreatic area was noted (Fig. 1C). From these findings, the patient was diagnosed with borderline resectable perihilar cholangiocarcinoma with lymph node metastasis and vascular invasion (Bismuth Type IIIa, UICC cT3N1M0 Stage IIIC).

**Figure 2:**
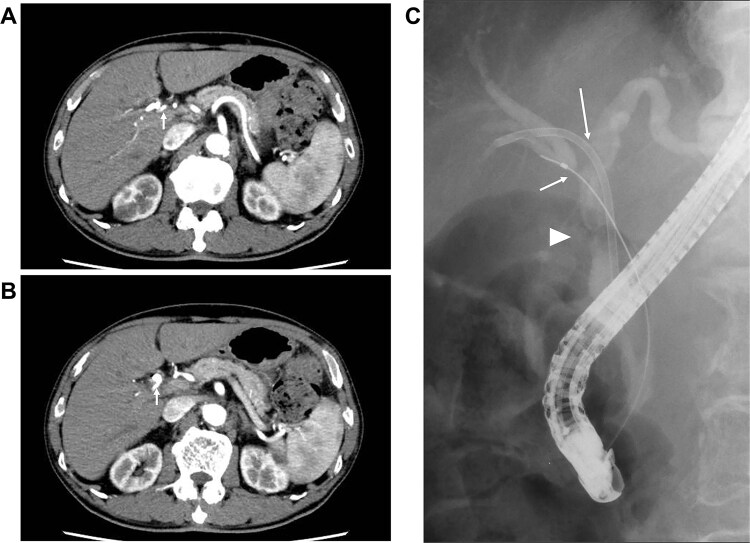
Images after seven cycles of GCD immunochemotherapy (A and B) dynamic CT demonstrating the tumor has shrunk and that the infiltration into the RHA has disappeared (arrow). The enlarged lymph node has also disappeared. (C) ERC revealing a significantly improved CHD stricture (arrow head); however, the stricture at the confluence of the anterior and posterior branches persists (arrow).

Seven cycles of GCD immunochemotherapy were performed (gemcitabine 1000 mg/m^2^ on days 1 and 8; cisplatin 25 mg/m^2^ on days 1 and 8; and durvalumab 1500 mg on day 1 in a 21-day cycle). During this period, GC administration on day 8 was discontinued in three of the seven cycles owing to granulocytopenia or decreased renal function, which was judged as Grade 2 according to Common Terminology Criteria for Adverse Events v5.0 [3]; however, durvalumab could be administered in all cycles. Dynamic CT performed after the seven cycles of GCD immunochemotherapy revealed significant tumor shrinkage and the disappearance of RHA invasion (Fig. 2A). Moreover, lymph node metastasis had disappeared (Fig. 2B). Post-chemotherapy ERC demonstrated markedly improved CHD narrowing; however, bile duct stenosis was noted at the confluence of the right anterior and posterior bile ducts (Fig. 2C). From these findings, efficacy assessment demonstrated a partial response, rendering the tumor resectable.

**Figure 3:**
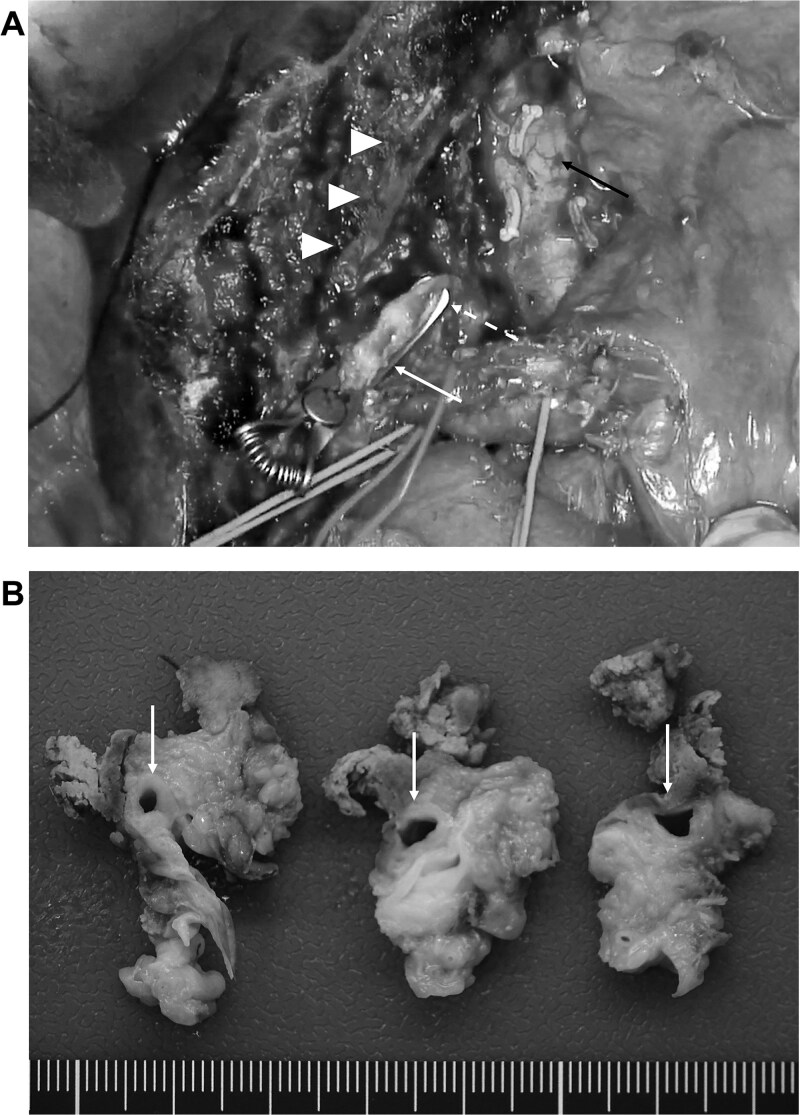
(A) Post-resection surgical field. The arrow head indicates the middle hepatic vein, and the white arrow, white dotted line, and black arrow indicate the stump of the right anterior bile duct, right posterior bile duct, and inferior vena cava, respectively. (B) Resected specimen revealing that the CHD has a clear border with the surrounding tissues; however, marked circumferential thickening and wall sclerosis are noted (arrow).

At 6 months following GCD immunochemotherapy initiation, left hepatectomy with caudate lobectomy, lymph node dissection, and hepaticojejunostomy were performed (Fig. 3A). The operative time was 546 min, and the estimated blood loss was 512 ml.

**Figure 4:**
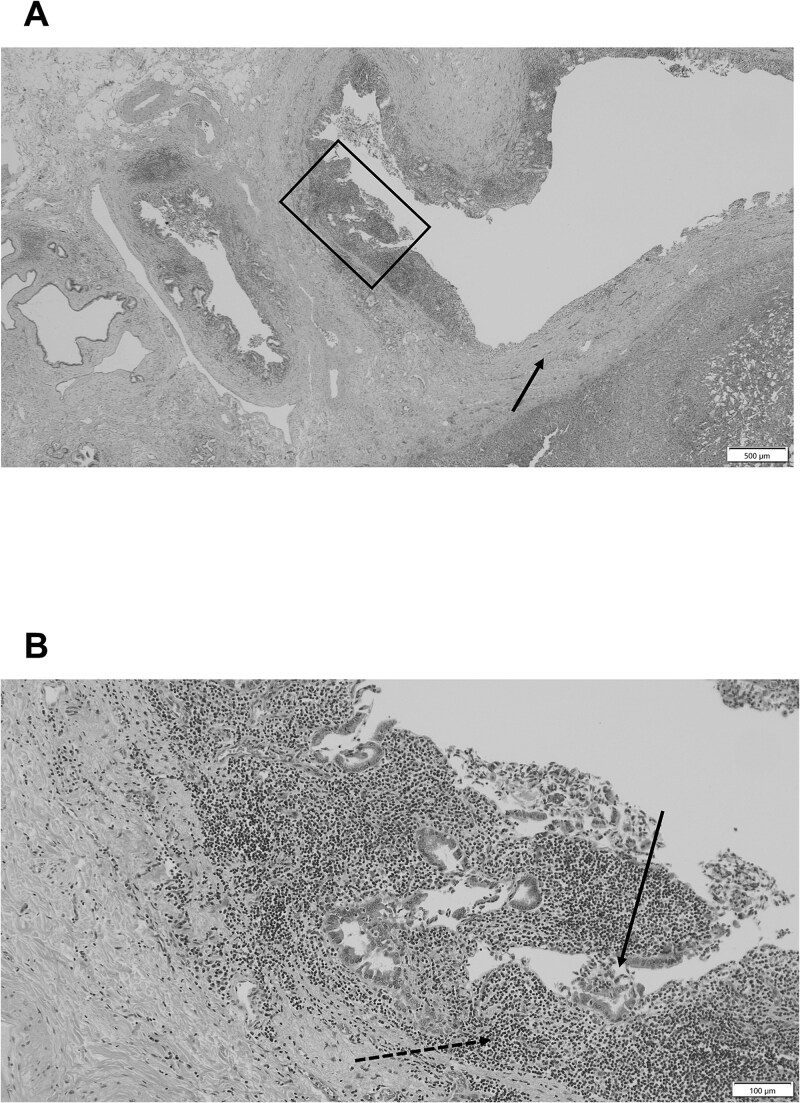
(A) Pathological examination revealing significant fibrosis of the bile duct wall and surrounding tissues (arrow). (B) Pathological examination of the enlarged square in a demonstrating marked inflammatory cell infiltration (dotted arrow) and reactive atypia (arrow) in the bile duct epithelium, and a lymphocyte and plasma cell infiltrate with fibrosis is noted around the bile ducts. No cancer cells are detected.

Macroscopically, the CHD exhibited a clear border; however, marked circumferential thickening and wall sclerosis were observed (Fig. 3B). Furthermore, the hepatoduodenal ligament showed inflammatory thickening; however, no evidence of extrahepatic bile duct invasion was noted. Pathological examination confirmed marked inflammatory cell infiltration and reactive atypia in the bile duct epithelium, and lymphocyte and plasma cell infiltrate with fibrosis was observed around the bile ducts (Fig. 4A and B). All sections showed no adenocarcinoma or lymph node metastasis, which was classified as Grade 4 according to the Evans classification [4]. An additional five cycles of GCD immunochemotherapy were administered postoperatively. At 2 years postoperatively, the patient is stable and shows no evidence of recurrence.

## Discussion

Vascular invasion and lymph node metastasis are well-established prognostic factors, even after curative surgery for perihilar cholangiocarcinoma [5–7]. Matsuyama *et al.* [8] also reported that the OS in patients with perihilar cholangiocarcinoma exhibiting lymph node metastasis and pathological vascular invasion was similar to that in patients with unresectable disease. Therefore, we planned to perform curative surgery once these risk factors were mitigated by GCD immunochemotherapy.

Historically, GC chemotherapy remained the standard of care for treating advanced or unresectable BTCs in the first-line setting for >10 years [5]. However, the prognosis for patients with advanced or unresectable BTCs remains poor. Recently, this treatment paradigm has shifted with the introduction of immune checkpoint inhibitors in addition to standard-of-care chemotherapy. Durvalmab is an immunoglobulin G antibody targeting programmed death-ligand 1 (PD-L1), inhibiting its interaction with PD-1, leading to cytotoxic T-cell disinhibition [9]. In 2022, the TOPAZ-1 trial was conducted, which was a double-blind placebo-controlled Phase 3 study, and reported that the mOS and median PFS (mPFS) of patients who received GCD immunochemotherapy (12.8 and 7.2 months, respectively) were significantly longer than those of patients who received GC therapy (11.5 and 5.7 months, respectively) [1]. Moreover, response rates improved with the addition of durvalumab (27% vs. 19%) to chemotherapy. These promising results rendered GCD immunochemotherapy the first-line treatment for advanced and unresectable BTCs. Subsequently, clinical trial and real-world data reported mOS of 10–16 months, mPFS of 5–8 months, and ORR of 11.1%–34.5% in patients who received GCD immunochemotherapy, which were superior to the outcomes obtained among those who received GC therapy [10–13]. CR rates were reported to be low (2.2%–4.5%); however, they were considered promising outcomes compared with those achieved by GC chemotherapy (0%–0.6%). Dong et al. [2] investigated neoadjuvant GCD immunochemotherapy in borderline resectable cholangiocarcinoma. Their results revealed that 46.2% (12/26) of the cases were converted to curative surgery and that 11 of the 12 resected patients were alive. The proportion of patients was very small; however, based on the findings, GCD immunochemotherapy may be considered promising for achieving a tumor reduction effect preoperatively, as demonstrated in our case. However, the high incidence of adverse events (AEs) should be considered; several studies have reported an incidence of Grade 3–4 AEs as high as 27%–75% [1, 10–13]. In our case, GC administration on day 8 was discontinued in three of the seven cycles owing to Grade 2 AEs; however, durvalumab could be administered in all cycles, potentially contributing to CR.

## Conclusion

GCD immunochemotherapy facilitated the downstaging of borderline resectable BTCs, including perihilar cholangiocarcinoma, and conversion surgery was successful in our case. To elucidate the efficacy and validity of multimodal therapeutic approaches, including conversion surgery, further trials and studies are warranted.
